# Case report: Coexistence of myotonia congenita and Brugada syndrome in one family

**DOI:** 10.3389/fneur.2022.1011956

**Published:** 2022-09-23

**Authors:** Ann Cordenier, Anja Flamez, Thomy de Ravel, Alexander Gheldof, Luigi Pannone, Carlo De Asmundis, Gudrun Pappaert, Véronique Bissay

**Affiliations:** ^1^Department of Neurology, Center for Neurosciences, Vrije Universiteit Brussel (VUB), UZ-Brussel, Brussels, Belgium; ^2^Center for Medical Genetics, Vrije Universiteit Brussel (VUB), UZ-Brussel, Brussels, Belgium; ^3^Heart Rhythm Management Centre, Vrije Universiteit Brussel (VUB), UZ-Brussel, Brussels, Belgium

**Keywords:** myotonia congenita, Brugada, cardiac arrhythmia, CLCN1, channelopathies

## Abstract

Myotonia congenita is a rare neuromuscular disorder caused by *CLCN1* mutations resulting in delayed muscle relaxation. Extramuscular manifestations are not considered to be present in chloride skeletal channelopathies, although recently some cardiac manifestations have been described. We report a family with autosomal dominant myotonia congenita and Brugada syndrome. Bearing in mind the previously reported cases of cardiac arrhythmias in myotonia congenita patients, we discuss the possible involvement of the CLCN1-gene mutations in primary cardiac arrhythmia.

## Introduction

Non-dystrophic myotonias (NDMs) are rare genetic neuromuscular disorders caused by dysfunctional ion channels expressed in the skeletal muscle cell membrane and, depending on the ion channel involved, referred to as chloride or sodium channelopathies.

Myotonia congenita is a chloride channelopathy with autosomal dominant or recessive inheritance, leading to Thomsen's or Becker's disease, respectively. Chloride channels (ClC-1), encoded by the *CLCN1* gene, play an essential role in restoring and maintaining the electrical stability of skeletal muscle cells. Up till now, more than 150 different *CLCN1* mutations, resulting in a reduced conductance of the ClC-1, have been described (1–3). Sodium channelopathies are caused by mutations in the *SCN4A* gene, resulting in an impaired inactivation of the alpha-subunit of the skeletal muscle NaV1.4 channel. Two autosomal dominant inherited phenotypes are recognized, paramyotonia congenita (PMC) and sodium channel myotonia (SCM) (1, 3). Both ion channelopathies lead to hyperexcitability of the sarcolemma membrane, causing myotonia, characterized by delayed skeletal muscle relaxation after voluntary or evoked muscle contraction. The first symptoms usually appear in the first or second decade but may also emerge later in life. MC is characterized by muscle stiffness (myotonia) upon initiating movement, and alleviated by repeated muscle contractions, known as the “warm-up” phenomenon. Transient muscle weakness after initiating movements as well as muscle hypertrophy can be part of the phenotype. Symptoms in Becker's disease are often more severe (4). In contrast to *SCN4A*-associated myotonia, face and hand muscles are less involved in MC, and no or minimal cold sensitivity is reported. However, distinguishing SCM from MC can be challenging since there is some clinical overlap (1). Diagnosis is made by medical history, physical examination, neurophysiological tests, and genetic analysis. Most NDM patients present myotonic discharges on needle electromyography (nEMG). In addition, Fournier's short exercise test (SET) can be performed to differentiate sodium and chloride channelopathies based on postexercise changes in compound muscle action potential (CMAP) amplitude obtained by supramaximal nerve stimulation. In most MC cases, the CMAP amplitude decreases instantaneously after a short effort and quickly returns to baseline, classified as pattern II (5). However, in a minority of autosomal dominant MC cases, the CMAP amplitude is not altered by exercise, resembling the pattern III characteristics of SCM (5).

NDMs are skeletal muscle disorders without extramuscular manifestations. However, in 2016 our study group reported four families with coexisting Brugada syndrome (BrS) and NDM. Three families were diagnosed with genetically confirmed SCM and one with autosomal dominant MC (Thomsen disease) (6). BrS is another rare inherited channelopathy, due to alterations of ion currents at the level of the cardiac sarcolemma, predisposing to life-threatening ventricular arrhythmias and sudden cardiac death (SCD). The estimated prevalence is 1 in 2000 people worldwide. The diagnosis is confirmed on spontaneous or Ajmaline-induced ST segment elevation on the electrocardiogram (ECG) with a type I morphology of > 2 mm in more than one lead among the right precordial leads (V1-V3) (7, 8). Approximately 20–30% of BrS cases are attributed to a mutation in the *SCN5A* gene. Other genes are assumed to be causative in an additional 10% of cases. However, most cases remain genetically unsolved. We hereby present a second family with MC and coexisting BrS.

## Case presentation

The index patient, a 52-year-old man (A1, Table 1), was referred to our neurology department. He complained of chronic painful muscle stiffness, mainly involving the shoulder and pelvic girdle and, to a lesser degree, the hands. He reported a warm-up phenomenon. Stress and preceding rest periods were exacerbating factors. Cold exposure, on the other hand, was not. He never experienced muscle weakness. These muscle complaints had been until now, attributed to fibromyalgia. Most importantly, there was a history of symptomatic BrS for which an ICD was implanted (Table 1). Upon physical examination diffuse hypertrophy was noted. When the patient tried to get up from a chair, initially we observed marked muscle stiffness of the lower limbs, which progressively improved upon repetition of the task. No other clinical myotonia was detected, including during the evaluation of hand grip, and eye closure. No percussion myotonia or lid lag could be elicited. Muscle strength was normal, and Gower's sign was negative. nEMG showed myotonic discharges mostly exceeding 1 second of duration in all muscles tested, without any myopathic features. The short exercise test was normal at room temperature and after cooling (pattern III). Genetic testing revealed that the patient was heterozygous carrier for a c.2287C>A, p.(Gln763Lys) variant in exon 19 of the *CLCN1* gene, which is classified as a variant of unknown significance (VUS). Other genetic causes of myotonia, including myotonic dystrophy type 1 (*DMPK*) and 2 (*ZNF9*) and sodium channelopathies (*SCN4A)*, were excluded. Subsequently we screened three relatives of our index patient, 2 of whom had BrS, for the presence of myotonia.

**Table 1:** Clinical and paraclinical features of the 4 relatives.

			**Neurological**				**Cardiological**						
**Patient**	**Age (Y)**	**Sex**	**Clinical findings**	**MD on** **nEMG**	**SET room temp/ cooling**	**Genetic tests**	**Clinical** **findings**	**Baseline** **ECG**	**Ajm**	**BrS** **diagnosis**	**EPS**	**Genetic** **result**	**ICD**
A1	53	M	MS, myalgiaWUP MH	+ All tested muscles	Type III/III	HZc of NM_000083.3(CLCN1): c.2287C>A, p.(Gln763Lys) SCN4A: WT DMPK: WT ZNF9: WT	Palpitations syncope	Nl	Type 1 pattern	Confirmed	Nl	SCN5A: WT BRSGP: WT	+
A2	60	F	MS, WUP	+ All tested muscles	Type III/III	HZc of NM_000083.3(CLCN1): c.2287C>A, p.(Gln763Lys) SCN4A: WT	aS	Nl	Nl	-No BrS diagnosis	NP	NP	-
A3	56	F	aSp	-*	Type III /III	CLCN1: WT SCN4A: WT DMPK: WT	Aborted SCD	Nl	Type 1 pattern	Confirmed	NP	SCN5A: WT	+
A4	24	M	aS	-	NP	CLCN1: WT	aS	Nl	Type 1 pattern	Confirmed	Nl	BRSGP: WT	-

Ajm, Ajmaline challenge test; aS, asymptomatic; aSp, aspecific muscular symptoms; BrS, Brugada syndrome; BRSGP, Brugada syndrome gene panel; DMPK, Myotonic Dystrophy Protein Kinase gene; ECG, electrocardiogram; EPS, electrophysiological study; F, female; HZc, Heterozygous carrier; ICD, implantable cardioverter defibrillator; M, male; MD, myotonic discharges; MH, muscle hypertrophy; MS, muscle stiffness; nEMG, needle-electromyography; Nl, Normal; NP, not-performed; SCD, sudden cardiac death; SET, short exercise test of Fournier; VUS, variant of unknown significance; WUP, warm-up phenomenon; WT, Wild Type; Y, Years.

* Muscle cramp in intrinsic hand muscle, not in the other tested muscles. +, present; −, absent.

**Figure 1:**
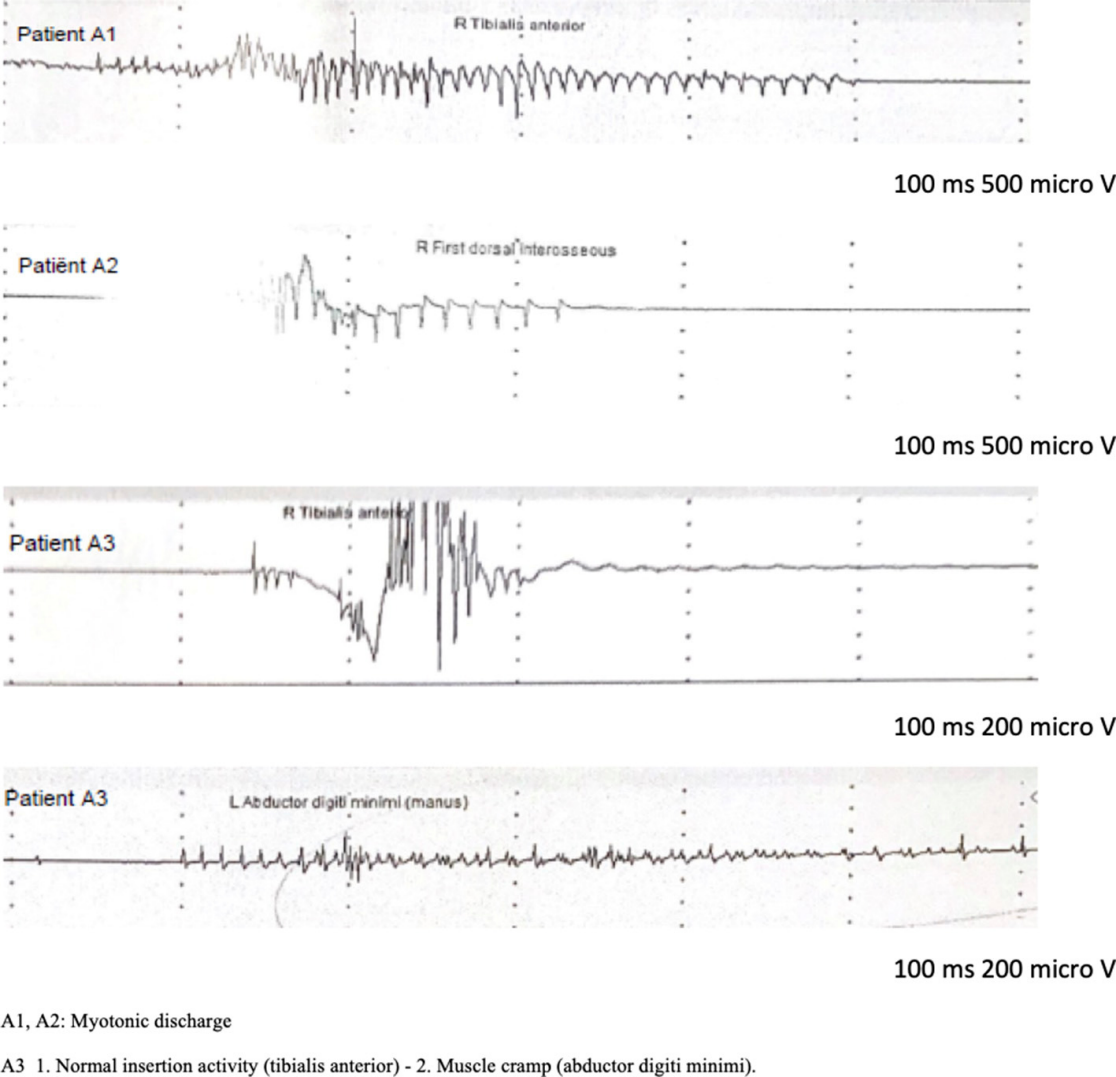
nEMG findings.

The eldest sister (A2) complained of muscle stiffness in the 4 limbs, especially after a period of rest, or after heavy exercise. There was no aggravation with exposure to cold. She had normal muscle strength and no signs of muscle hypertrophy. Although no clinical myotonia was detected, nEMG did reveal brief (100–200 msec duration) myotonic discharges in several muscles. The short exercise test was normal at room temperature and after cooling (pattern III). Sequencing the *CLCN1* gene, the same heterozygous c.2287C>A, p.(Gln763Lys) variant in exon 19 was detected. No Brugada syndrome was diagnosed since she had a negative Ajmaline challenge test. The second sister (A3) had been diagnosed with Brugada Syndrome following a malignant cardiac event requiring an ICD implantation. She did not complain of muscle stiffness, myalgia, or delayed muscle relaxation. She mentioned sporadic muscle cramps, not preventing her from exercising. The neurological exam was normal, and no muscle hypertrophy nor clinical myotonia was present. During nEMG no myotonic discharges were observed. However, a slightly prolonged random insertional activity, was found in the intrinsic hand muscles and not in the other tested muscles, including the tibial anterior and biceps brachii. These findings were interpreted as a cramp and not as myotonic discharges. This was supported by the fact that at the same time, the patient experienced a slight painful cramping sensation at the site of the needle insertion. A short exercise test was also normal at room temperature and after cooling of the investigated muscle. Based on these clinical and electrophysiological features, no myotonic phenotype was retained. In the context of genetic screening of this family, the analysis of *CLCN1, SCN4A*, and *DMPK* genes was normal in this patient (A3). The son of our index patient A1 (A4) had a first negative Ajmaline challenge test at the age of 11 which turned positive when he was 19 years old, confirming the diagnosis of BrS. He never had any cardiac or neurological symptoms. No clinical or electrical abnormalities were detected during physical examination and nEMG. His genetic screening showed no variants in the *CLCN1* gene. No *SCN5A* variant was found in this family. A primary cardiac arrhythmia gene panel was performed of the index patient (A1) and his son (A4). They both returned normal. The following genes were tested in this panel: ANK3, CACNA1c, CACNA2D1, CACNB2b, GPD1L, HCN4, KCNA3, KCNA4, KCNA7, KCNC4, KCND2, KCND3, KCNE1L (KCNE5), KCNE3, KCNH2, KCNJ8, RANGRF (MOG1), SCN1B, SCN2B, SCN3B, SCN5A, SCN7A (Nav.2.1), SGK1, SGK3, SLMAP, SNTG2, TRPM4. The clinical and paraclinical features of the 4 relatives are summarized in Table 1. The nEMG findings are shown in Figure 1.

## Discussion

We report a family with a clinical and electrophysiological phenotype of NDM and the presence of a heterozygous c.2287C>A (p.Gln763Lys) *CLCN1* gene variant. Although this variant was previously reported as likely benign by Brugnoni et al. we believe that in our family this variant is probably pathogenic (13). Indeed, Thomsen's disease is the most probable diagnosis based on the presence of cold-insensitive predominant lower limb muscle stiffness and associated warm-up phenomenon, muscle hypertrophy, myotonic discharges without myopathic features on nEMG, further supported by segregation analysis of this pedigree, and the absence of other gene mutations related to familial myotonic syndromes, including *SCN4A, DMPK*, and *ZNF9* (14–16). Some family members of this pedigree were previously diagnosed with BrS. *SCN5A* sequencing and the primary cardiac arrhythmia panel analysis were normal. However, a causative role of the detected *CLCN1* variant in relation to the arrythmia can be ruled out since two relatives (A3, A4) were diagnosed with BrS and did not carry this *CLCN1* variant.

In 2016, our study group reported the first large family with Thomsen's disease due to a novel described *CLCN1* mutation and coexisting BrS. Thirteen relatives with BrS were reported, of whom 11 carried the NM_198056.3(SCN5A): c.2632C>T, p.(Arg878Cys) variant of unknown significance (VUS), and five had a genetically confirmed MC due to the NM_000083.3(CLCN1): c.744+1G>A;[=] pathogenic variant. Remarkably, two relatives who showed a positive Ajmaline challenge test without a history of malignant cardiac events, carried the *CLCN1* variant and not the *SCN5A* VUS (6).

Despite the absence of similar cases in the literature reporting the coexistence of BrS and MC, other cardiac arrhythmias and conduction disturbances have recently been described in patients with MC. Vereb et al. reported six MC patients who exhibited arrhythmias or conduction disorders, some of whom requiring a pacemaker (9). Wang et al. described nine autosomal dominant and recessive MC patients with cardiac arrhythmia resulting in minor cardiac symptoms. However, genetic analyses exploring primary cardiac arrhythmias were not specified in this article (10). Two additional case reports described the coexistence of MC and cardiac arrhythmia, including Wolff-Parkinson-White syndrome (11, 12). An overview of reported cases of cardiac arrhythmias in myotonia congenita patients is given in Table 2.

**Table 2:** Reported cases of cardiac arrhythmias in myotonia congenita patients.

**Study**	**N**	**Muscular features**	**Cardiac features**
Bissay et al. (6)	5	none, sporadic muscle stiffness at the thumb, sporadic cramps calf at night	+ Ajmaline test in 5 patients: asymptomatic, RBBB, VF
Vereb et al. (9)	48	Myotonia, myalgia, cramps, paresis, muscle hypertrophy	6 of 48 patients: RBBB (2), cardiac arrhythmia (2), AV-block II, AV-block III, pacemaker implantation (3)
Wang et al. (10)	17	Muscle stiffness, muscle hypertrophy, warm-up phenomenon	9 of 17 patients: Sinus tachycardia (2), atrial premature beats (2), ventricular premature beats (2), sinus arrythmia (2)
Caballero (11)	1	Myotonia, transient weakness, muscular hypertrophy	Wolf-Parkinson-White-Syndrome
Anderson (12)	1	Myotonia of grip and percussion	AV- block II with Wenckebach conduction disturbance

It is unclear whether *CLCN1* mutations may contribute to primary arrhythmias or conduction disorders. The ClC-1 channels are mainly expressed in the skeletal muscle, but low levels are also found in the kidney, heart, liver, smooth muscle, and the central nervous system (16–18). Other chloride channel isoforms, including ClC-2 and ClC-3 voltage-gated chloride channels, have been implicated in cardiac arrhythmias (19). By contrast, no clear link has been reported between cardiac ClC-1 channels and cardiac arrhythmias, including BrS. However, for several reasons our clinical data may support a potential role of *CLCN1* in the pathogenesis of cardiac arrhythmia, especially BrS. First, since the likelihood of cardiac and muscular channelopathies occurring together in patients is extremely low, given their rare prevalence in the general population, a pathophysiological link between *CLCN1* mutations and cardiac arrhythmias can be suspected. Second, we can assume that the prevalence of *CLCN1*-associated myotonia is underestimated in the BrS population, due to the rather unspecific symptoms, especially in mild cases. Patients may complain of chronic muscle stiffness and myalgia without muscle weakness. Because those symptoms are unspecific and common, NDM is rarely suspected, and diagnosis is often delayed. This is clearly illustrated by the history of our family cases A1 and A2, where in the former symptoms were attributed to an earlier fibromyalgia diagnosis, and, in the latter, mild muscular symptoms were not even reported. Thanks to increasing awareness of the presence of subtle muscular symptoms in myotonia, the rhythmology team referred the BrS patient (A1) to our neurology department. The NDM diagnosis would probably not yet have been made in other circumstances. Finally, the Mendelian model for BrS, characterized by single gene disease, has been recently challenged by an oligogenic model referring to a possible cumulative role of common and rare genetic variants (20). A possible explanation could be that *CLCN1* variants contribute to the BrS phenotype. On the other hand, it cannot be excluded that it is a simple coexistence of two separate pathologies and that the gene responsible for Brugada syndrome in these patients is not yet known.

A parallel can be drawn with the *SCN4A* gene recently added to the list of minor genes associated with BrS (7, 8). In 2016, our study group reported 3 families with *SCN4A*-associated NDM and coexisting BrS, with this gene presumably acting as a modifier gene. Some of these patients had few muscle complaints, despite the presence of a deleterious variant in the *SCN4A* gene (6). The expression of the skeletal *SCN4A* isoform in cardiac muscle was also described (21, 22).

Even though current data are not sufficient to advocate a systematic cardiac work-up in patients with NDM, there is increasing evidence of an overlap between skeletal muscle and cardiac ion channelopathies. Therefore, we propose to perform at least a thorough cardiac history assessment and a 12-lead ECG in NDM patients. A history of palpitations, syncopes, conduction disorders, or a family history of sudden cardiac death should prompt an immediate referral to a cardiologist. Conversely, patients with primary cardiac arrhythmias, especially those with BrS, should be systematically asked about presence of muscle stiffness, myalgia, muscle weakness, and if indicated, be referred to the neurology department.

Sodium channel blockers are used in the symptomatic treatment of myotonia. Mexiletine, a class 1B antiarrhythmic drug, seems to be a safe anti-myotonic therapy in the presence of Brugada syndrome (23, 24). However, the use of Flecainide, a class 1C antiarrhythmic drug, should be started with caution, based on a case report in which BrS was unmasked after initiating this treatment in an SCM patient who had never experienced cardiac symptoms before (25). Considering this and the previously reported family with concomitant MC and BrS, the use of class 1C antiarrhythmic drug to alleviate myotonia seems less appropriate for MC patients as well (6).

Of course, our data need to be confirmed by new designed clinical and genome wide association studies in larger patient cohorts, as well as functional studies in order to further clarify the involvement of *CLCN1* gene in cardiac arrhythmias.

## Data availability statement

The datasets presented in this article are not readily available because of ethical and privacy restrictions. Requests to access the datasets should be directed to the corresponding author.

## Ethics statement

Written informed consent was obtained from the individual(s) for the publication of any potentially identifiable images or data included in this article.

## Author contributions

AC: data collection, review of the literature, and article writing. VB: review of literature, article writing, and article editing. AF, TR, AG, LP, CD, and GP: article editing. All authors contributed to the article and approved the submitted version.

## Conflict of interest

The authors declare that the research was conducted in the absence of any commercial or financial relationships that could be construed as a potential conflict of interest.

## Publisher's note

All claims expressed in this article are solely those of the authors and do not necessarily represent those of their affiliated organizations, or those of the publisher, the editors and the reviewers. Any product that may be evaluated in this article, or claim that may be made by its manufacturer, is not guaranteed or endorsed by the publisher.
